# Crystal structure of benzobi­cyclon

**DOI:** 10.1107/S2056989015023221

**Published:** 2015-12-09

**Authors:** Gihaeng Kang, Jineun Kim, Hansu Lim, Tae Ho Kim

**Affiliations:** a Department of Chemistry and Research Institute of Natural Sciences, Gyeongsang National University, Jinju 52828, Republic of Korea

**Keywords:** crystal structure, benzobi­cyclon, bi­cyclo­[3.2.1]oct-2-en-4-one, herbicide

## Abstract

In the title compound, C_22_H_19_ClO_4_S_2_ [systematic name: 3-(2-chloro-4-mesylbenzo­yl)-4-(phenyl­sulfan­yl)bi­cyclo­[3.2.1]oct-3-en-2-one], which is an unclassified herbicide, the dihedral angle between the plane of the phenyl and chloro­benzene rings is 19.9 (2)°. In the crystal, C—H⋯O hydrogen bonds link adjacent mol­ecules, generating two-dimensional networks extending parellel to (011).

## Related literature   

For information on the herbicidal properties of the title compound, see: Im *et al.* (2015 ▸). For related crystal structures, see: Brown *et al.* (2007 ▸); Hou *et al.* (2010 ▸).
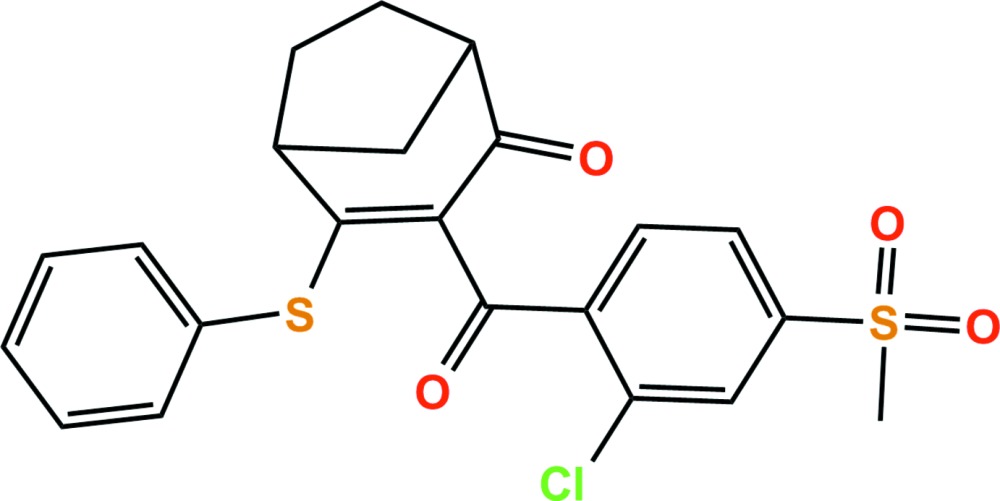


## Experimental   

### Crystal data  

C_22_H_19_ClO_4_S_2_
*M* *_r_* = 446.94
Monoclinic, 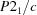

*a* = 15.5111 (8) Å
*b* = 10.7218 (6) Å
*c* = 12.0169 (6) Å
β = 91.449 (3)°
*V* = 1997.85 (18) Å^3^
*Z* = 4
Mo *K*α radiation
μ = 0.43 mm^−1^
*T* = 173 K
0.15 × 0.11 × 0.06 mm

### Data collection  

Bruker APEXII CCD diffractometer
Absorption correction: multi-scan (*SADABS*; Bruker, 2014 ▸) *T*_min_ = 0.687, *T*_max_ = 0.746
15850 measured reflections
3504 independent reflections
2584 reflections with *I* > 2σ(*I*)
*R*_int_ = 0.055

### Refinement  

*R*[*F* ^2^ > 2σ(*F* ^2^)] = 0.053
*wR*(*F* ^2^) = 0.142
*S* = 1.07
3504 reflections
263 parameters
H-atom parameters constrained
Δρ_max_ = 0.97 e Å^−3^
Δρ_min_ = −0.54 e Å^−3^

### 

Data collection: *APEX2* (Bruker, 2014 ▸); cell refinement: *SAINT* (Bruker, 2014 ▸); data reduction: *SAINT*; program(s) used to solve structure: *SHELXS97* (Sheldrick, 2008 ▸); program(s) used to refine structure: *SHELXL2014* (Sheldrick, 2015 ▸); molecular graphics: *DIAMOND* (Brandenburg, 2010 ▸); software used to prepare material for publication: *SHELXTL* (Sheldrick, 2008 ▸).

## Supplementary Material

Crystal structure: contains datablock(s) global, I. DOI: 10.1107/S2056989015023221/sj5485sup1.cif

Structure factors: contains datablock(s) I. DOI: 10.1107/S2056989015023221/sj5485Isup2.hkl

Supporting information file. DOI: 10.1107/S2056989015023221/sj5485Isup3.cml

**Fig. 1.**
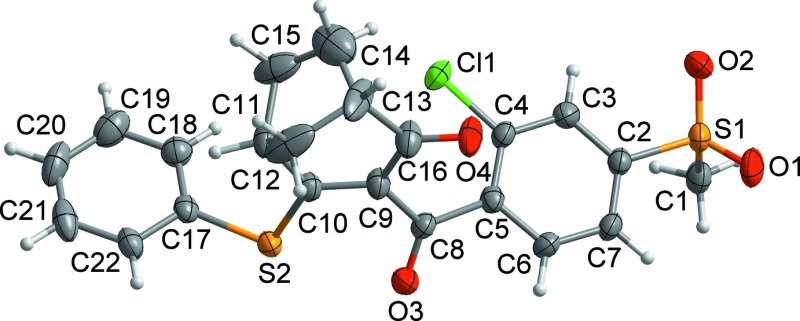
The asymmetric unit of the title compound with the atom-numbering scheme. Displacement ellipsoids are drawn at the 50% probability level. H atoms are shown as small spheres of arbitrary radius.

**Fig. 2.**
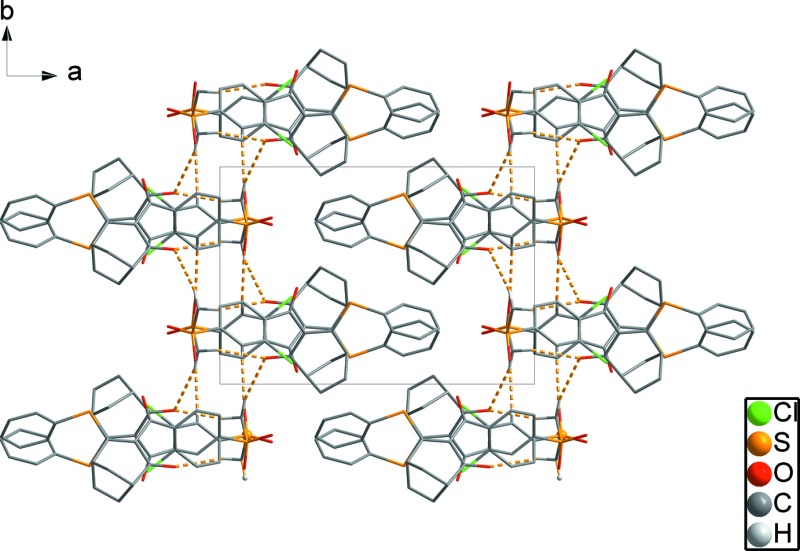
Crystal packing viewed along the c axis. The intermolecular hydrogen bonds are shown as dashed lines.

. DOI: 10.1107/S2056989015023221/sj5485fig1.tif
The asymmetric unit of the title compound with the atom-numbering scheme. Displacement ellipsoids are drawn at the 50% probability level. H atoms are shown as small spheres of arbitrary radius.

c . DOI: 10.1107/S2056989015023221/sj5485fig2.tif
Crystal packing viewed along the *c* axis. The inter­molecular hydrogen bonds are shown as dashed lines.

CCDC reference: 1440215

Additional supporting information:  crystallographic information; 3D view; checkCIF report

## Figures and Tables

**Table 1:** Hydrogen-bond geometry (Å, °)

*D*—H⋯*A*	*D*—H	H⋯*A*	*D*⋯*A*	*D*—H⋯*A*
C1—H1*B*⋯O4^i^	0.98	2.44	3.322 (4)	150
C1—H1*C*⋯O4^ii^	0.98	2.52	3.477 (5)	165
C3—H3⋯O2^iii^	0.95	2.53	3.422 (4)	157

Symmetry codes: (i) 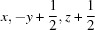; (ii) 
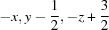
; (iii) 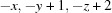.

## supplementary crystallographic information

### S1. Comment

Benzobicyclon is a newly developed compound with potent herbicidal activity against Scirpus juncoides, one of the most troublesome weeds in paddy fields. (Im *et al.*, 2015). However, until now its crystal structure has not been reported. In the title compound (Fig. 1), the dihedral angle between the plane of the phenyl and chlorobenzene rings is 19.9 (2)°. All bond lengths and bond angles are normal and comparable to those observed in the crystal structures of similar compounds (Brown *et al.*, 2007; Hou *et al*., 2010).

In the crystal structure (Fig. 2), intermolecular C1—H1B···O4 hydrogen bond link adjacent molecules, forming chains along the *c*-axis direction. These chains are linked by C1—H1C···O4 and C3—H3···O2 hydrogen bonds (Table 1), resulting in two-dimensional networks extending parellel to the (011) plane.

### S2. Experimental

The title compound was purchased from the Dr. Ehrenstorfer GmbH Company. Slow evaporation of a solution in CH_2_C1_2_ gave single crystals suitable for X-ray analysis.

### S3. Refinement

All H-atoms were positioned geometrically and refined using a riding model with d(C—H) = 1.00 Å, *U*_iso_ = 1.2*U*_eq_(C) for C*sp*^3^—H, d(C—H) = 0.99 Å, *U*_iso_ = 1.2*U*_eq_(C) for CH_2_ groups, d(C—H) = 0.98 Å, *U*_iso_ = 1.2*U*_eq_(C) for CH_3_ groups, d(C—H) = 0.95 Å, *U*_iso_ = 1.2*U*_eq_(C) for aromatic C—H.

### Crystal data

**Table:**

C_22_H_19_ClO_4_S_2_	*F*(000) = 928
*M**_r_* = 446.94	*D*_x_ = 1.486 Mg m^−^^3^
Monoclinic, *P*2_1_/*c*	Mo *K*α radiation, λ = 0.71073 Å
*a* = 15.5111 (8) Å	Cell parameters from 2645 reflections
*b* = 10.7218 (6) Å	θ = 2.6–24.7°
*c* = 12.0169 (6) Å	µ = 0.43 mm^−^^1^
β = 91.449 (3)°	*T* = 173 K
*V* = 1997.85 (18) Å^3^	Block, colourless
*Z* = 4	0.15 × 0.11 × 0.06 mm

### Data collection

**Table:**

Bruker APEXII CCD diffractometer	2584 reflections with *I* > 2σ(*I*)
φ and ω scans	*R*_int_ = 0.055
Absorption correction: multi-scan (*SADABS*; Bruker, 2014)	θ_max_ = 25.0°, θ_min_ = 2.3°
*T*_min_ = 0.687, *T*_max_ = 0.746	*h* = −18→18
15850 measured reflections	*k* = −11→12
3504 independent reflections	*l* = −14→12

### Refinement

**Table:**

Refinement on *F*^2^	0 restraints
Least-squares matrix: full	Hydrogen site location: inferred from neighbouring sites
*R*[*F*^2^ > 2σ(*F*^2^)] = 0.053	H-atom parameters constrained
*wR*(*F*^2^) = 0.142	*w* = 1/[σ^2^(*F*_o_^2^) + (0.0619*P*)^2^ + 2.0045*P*] where *P* = (*F*_o_^2^ + 2*F*_c_^2^)/3
*S* = 1.07	(Δ/σ)_max_ < 0.001
3504 reflections	Δρ_max_ = 0.97 e Å^−^^3^
263 parameters	Δρ_min_ = −0.54 e Å^−^^3^

### Special details

**Table:**

Geometry. All e.s.d.'s (except the e.s.d. in the dihedral angle between two l.s. planes) are estimated using the full covariance matrix. The cell e.s.d.'s are taken into account individually in the estimation of e.s.d.'s in distances, angles and torsion angles; correlations between e.s.d.'s in cell parameters are only used when they are defined by crystal symmetry. An approximate (isotropic) treatment of cell e.s.d.'s is used for estimating e.s.d.'s involving l.s. planes.

### Fractional atomic coordinates and isotropic or equivalent isotropic displacement parameters (Å^2^)

**Table:**

	*x*	*y*	*z*	*U*_iso_*/*U*_eq_	
Cl1	0.22882 (6)	0.40049 (9)	0.83242 (9)	0.0464 (3)	
S1	−0.08667 (6)	0.26508 (9)	0.95779 (9)	0.0361 (3)	
S2	0.41445 (6)	0.12501 (8)	0.64532 (8)	0.0324 (3)	
O1	−0.16416 (16)	0.2346 (3)	0.8963 (2)	0.0495 (8)	
O2	−0.07793 (17)	0.3867 (2)	1.0065 (3)	0.0499 (8)	
O3	0.24478 (16)	0.0592 (2)	0.6713 (2)	0.0429 (7)	
O4	0.14394 (16)	0.3801 (3)	0.5647 (3)	0.0479 (8)	
C1	−0.0691 (2)	0.1524 (4)	1.0616 (3)	0.0401 (10)	
H1A	−0.1083	0.1674	1.1228	0.060*	
H1B	−0.0093	0.1576	1.0895	0.060*	
H1C	−0.0799	0.0692	1.0304	0.060*	
C2	0.0015 (2)	0.2413 (3)	0.8691 (3)	0.0303 (8)	
C3	0.0716 (2)	0.3211 (3)	0.8792 (3)	0.0339 (9)	
H3	0.0715	0.3884	0.9305	0.041*	
C4	0.1417 (2)	0.3002 (3)	0.8126 (3)	0.0309 (8)	
C5	0.1442 (2)	0.2011 (3)	0.7389 (3)	0.0290 (8)	
C6	0.0730 (2)	0.1218 (3)	0.7314 (3)	0.0335 (9)	
H6	0.0736	0.0530	0.6816	0.040*	
C7	0.0013 (2)	0.1420 (3)	0.7954 (3)	0.0338 (9)	
H7	−0.0473	0.0883	0.7890	0.041*	
C8	0.2214 (2)	0.1671 (3)	0.6715 (3)	0.0302 (8)	
C9	0.2696 (2)	0.2642 (3)	0.6103 (3)	0.0336 (9)	
C10	0.3565 (2)	0.2546 (3)	0.5982 (3)	0.0310 (8)	
C11	0.4045 (2)	0.3595 (4)	0.5419 (3)	0.0382 (9)	
H11	0.4656	0.3361	0.5274	0.046*	
C12	0.3542 (4)	0.3935 (5)	0.4330 (3)	0.0668 (16)	
H12A	0.3347	0.3191	0.3906	0.080*	
H12B	0.3870	0.4499	0.3845	0.080*	
C13	0.2767 (3)	0.4631 (4)	0.4939 (5)	0.0711 (17)	
H13	0.2407	0.5144	0.4411	0.085*	
C14	0.3207 (4)	0.5376 (5)	0.5754 (5)	0.0755 (17)	
H14A	0.2838	0.5499	0.6405	0.091*	
H14B	0.3341	0.6204	0.5438	0.091*	
C15	0.3992 (3)	0.4753 (4)	0.6092 (4)	0.0622 (14)	
H15A	0.3983	0.4548	0.6895	0.075*	
H15B	0.4495	0.5295	0.5958	0.075*	
C16	0.2224 (2)	0.3702 (4)	0.5594 (4)	0.0466 (11)	
C17	0.5234 (2)	0.1795 (3)	0.6555 (3)	0.0299 (8)	
C18	0.5496 (3)	0.2579 (3)	0.7411 (3)	0.0404 (10)	
H18	0.5085	0.2892	0.7914	0.048*	
C19	0.6355 (3)	0.2910 (4)	0.7538 (4)	0.0532 (12)	
H19	0.6536	0.3460	0.8118	0.064*	
C20	0.6948 (3)	0.2430 (4)	0.6807 (4)	0.0566 (13)	
H20	0.7540	0.2643	0.6896	0.068*	
C21	0.6690 (3)	0.1654 (4)	0.5962 (4)	0.0488 (12)	
H21	0.7103	0.1336	0.5465	0.059*	
C22	0.5831 (2)	0.1329 (3)	0.5825 (3)	0.0374 (9)	
H22	0.5652	0.0791	0.5236	0.045*	

### Atomic displacement parameters (Å^2^)

**Table:**

	*U*^11^	*U*^22^	*U*^33^	*U*^12^	*U*^13^	*U*^23^
Cl1	0.0325 (5)	0.0437 (6)	0.0632 (7)	−0.0172 (4)	0.0084 (5)	−0.0109 (5)
S1	0.0220 (5)	0.0359 (5)	0.0509 (6)	−0.0007 (4)	0.0085 (4)	−0.0012 (4)
S2	0.0273 (5)	0.0273 (5)	0.0430 (6)	0.0021 (4)	0.0086 (4)	0.0007 (4)
O1	0.0215 (13)	0.0641 (19)	0.063 (2)	0.0002 (12)	−0.0001 (13)	−0.0002 (15)
O2	0.0421 (16)	0.0354 (15)	0.073 (2)	−0.0006 (12)	0.0196 (15)	−0.0119 (14)
O3	0.0372 (15)	0.0298 (14)	0.0624 (19)	−0.0013 (12)	0.0170 (14)	−0.0004 (13)
O4	0.0221 (14)	0.0543 (17)	0.067 (2)	0.0025 (12)	0.0014 (13)	0.0214 (15)
C1	0.030 (2)	0.046 (2)	0.045 (2)	−0.0030 (17)	0.0105 (18)	0.0022 (19)
C2	0.0229 (18)	0.0305 (18)	0.038 (2)	−0.0010 (14)	0.0032 (16)	0.0033 (16)
C3	0.0296 (19)	0.0289 (18)	0.043 (2)	−0.0007 (15)	0.0015 (17)	−0.0017 (17)
C4	0.0235 (17)	0.0294 (18)	0.040 (2)	−0.0053 (15)	0.0033 (16)	0.0032 (16)
C5	0.0254 (18)	0.0278 (18)	0.034 (2)	0.0000 (14)	0.0047 (16)	0.0064 (16)
C6	0.032 (2)	0.0277 (18)	0.041 (2)	−0.0078 (15)	0.0045 (17)	−0.0026 (17)
C7	0.0230 (18)	0.0325 (19)	0.046 (2)	−0.0066 (15)	0.0019 (17)	0.0022 (17)
C8	0.0258 (18)	0.0282 (19)	0.037 (2)	−0.0045 (15)	0.0044 (16)	0.0028 (16)
C9	0.0239 (18)	0.036 (2)	0.041 (2)	0.0005 (15)	0.0052 (16)	0.0093 (17)
C10	0.0284 (19)	0.0329 (19)	0.032 (2)	0.0003 (15)	0.0050 (16)	0.0034 (16)
C11	0.0225 (18)	0.044 (2)	0.049 (2)	0.0000 (16)	0.0069 (17)	0.0151 (19)
C12	0.107 (4)	0.068 (3)	0.026 (2)	−0.048 (3)	0.012 (3)	0.002 (2)
C13	0.026 (2)	0.044 (3)	0.141 (5)	0.0005 (19)	−0.023 (3)	0.043 (3)
C14	0.095 (4)	0.061 (3)	0.072 (4)	0.008 (3)	0.031 (3)	0.018 (3)
C15	0.094 (4)	0.047 (3)	0.046 (3)	−0.030 (3)	0.011 (3)	0.000 (2)
C16	0.029 (2)	0.048 (2)	0.063 (3)	0.0016 (18)	0.005 (2)	0.020 (2)
C17	0.0255 (18)	0.0270 (18)	0.037 (2)	0.0048 (14)	0.0025 (16)	0.0080 (16)
C18	0.040 (2)	0.037 (2)	0.044 (2)	0.0001 (17)	0.0027 (19)	0.0038 (19)
C19	0.049 (3)	0.055 (3)	0.055 (3)	−0.011 (2)	−0.012 (2)	0.009 (2)
C20	0.027 (2)	0.068 (3)	0.074 (3)	−0.009 (2)	−0.007 (2)	0.029 (3)
C21	0.033 (2)	0.056 (3)	0.058 (3)	0.016 (2)	0.013 (2)	0.028 (2)
C22	0.031 (2)	0.038 (2)	0.044 (2)	0.0097 (17)	0.0096 (18)	0.0064 (18)

### Geometric parameters (Å, º)

**Table:**

Cl1—C4	1.739 (3)	C11—C15	1.485 (6)
S1—O1	1.432 (3)	C11—C12	1.551 (6)
S1—O2	1.434 (3)	C11—H11	1.0000
S1—C1	1.753 (4)	C12—C13	1.607 (7)
S1—C2	1.774 (3)	C12—H12A	0.9900
S2—C10	1.742 (4)	C12—H12B	0.9900
S2—C17	1.790 (4)	C13—C14	1.425 (8)
O3—C8	1.213 (4)	C13—C16	1.534 (6)
O4—C16	1.226 (4)	C13—H13	1.0000
C1—H1A	0.9800	C14—C15	1.437 (7)
C1—H1B	0.9800	C14—H14A	0.9900
C1—H1C	0.9800	C14—H14B	0.9900
C2—C7	1.385 (5)	C15—H15A	0.9900
C2—C3	1.386 (5)	C15—H15B	0.9900
C3—C4	1.384 (5)	C17—C18	1.382 (5)
C3—H3	0.9500	C17—C22	1.385 (5)
C4—C5	1.385 (5)	C18—C19	1.383 (6)
C5—C6	1.395 (5)	C18—H18	0.9500
C5—C8	1.508 (5)	C19—C20	1.387 (6)
C6—C7	1.384 (5)	C19—H19	0.9500
C6—H6	0.9500	C20—C21	1.365 (7)
C7—H7	0.9500	C20—H20	0.9500
C8—C9	1.487 (5)	C21—C22	1.383 (6)
C9—C10	1.363 (5)	C21—H21	0.9500
C9—C16	1.476 (5)	C22—H22	0.9500
C10—C11	1.517 (5)		
			
O1—S1—O2	119.16 (18)	C11—C12—C13	95.2 (3)
O1—S1—C1	108.84 (18)	C11—C12—H12A	112.7
O2—S1—C1	108.9 (2)	C13—C12—H12A	112.7
O1—S1—C2	107.89 (18)	C11—C12—H12B	112.7
O2—S1—C2	108.04 (16)	C13—C12—H12B	112.7
C1—S1—C2	102.72 (17)	H12A—C12—H12B	110.2
C10—S2—C17	103.94 (16)	C14—C13—C16	105.7 (5)
S1—C1—H1A	109.5	C14—C13—C12	102.8 (4)
S1—C1—H1B	109.5	C16—C13—C12	111.0 (3)
H1A—C1—H1B	109.5	C14—C13—H13	112.2
S1—C1—H1C	109.5	C16—C13—H13	112.2
H1A—C1—H1C	109.5	C12—C13—H13	112.2
H1B—C1—H1C	109.5	C13—C14—C15	108.8 (4)
C7—C2—C3	121.3 (3)	C13—C14—H14A	109.9
C7—C2—S1	120.4 (3)	C15—C14—H14A	109.9
C3—C2—S1	118.3 (3)	C13—C14—H14B	109.9
C4—C3—C2	118.4 (3)	C15—C14—H14B	109.9
C4—C3—H3	120.8	H14A—C14—H14B	108.3
C2—C3—H3	120.8	C14—C15—C11	107.1 (4)
C3—C4—C5	121.9 (3)	C14—C15—H15A	110.3
C3—C4—Cl1	116.1 (3)	C11—C15—H15A	110.3
C5—C4—Cl1	121.8 (3)	C14—C15—H15B	110.3
C4—C5—C6	118.3 (3)	C11—C15—H15B	110.3
C4—C5—C8	124.4 (3)	H15A—C15—H15B	108.6
C6—C5—C8	117.2 (3)	O4—C16—C9	121.8 (3)
C7—C6—C5	120.9 (3)	O4—C16—C13	121.9 (4)
C7—C6—H6	119.5	C9—C16—C13	116.1 (3)
C5—C6—H6	119.5	C18—C17—C22	120.2 (3)
C6—C7—C2	119.1 (3)	C18—C17—S2	120.6 (3)
C6—C7—H7	120.4	C22—C17—S2	119.0 (3)
C2—C7—H7	120.4	C17—C18—C19	120.1 (4)
O3—C8—C9	120.9 (3)	C17—C18—H18	119.9
O3—C8—C5	118.2 (3)	C19—C18—H18	119.9
C9—C8—C5	120.9 (3)	C18—C19—C20	119.2 (4)
C10—C9—C16	119.7 (3)	C18—C19—H19	120.4
C10—C9—C8	120.7 (3)	C20—C19—H19	120.4
C16—C9—C8	119.6 (3)	C21—C20—C19	120.8 (4)
C9—C10—C11	119.4 (3)	C21—C20—H20	119.6
C9—C10—S2	121.9 (3)	C19—C20—H20	119.6
C11—C10—S2	118.7 (3)	C20—C21—C22	120.3 (4)
C15—C11—C10	110.0 (3)	C20—C21—H21	119.9
C15—C11—C12	103.2 (4)	C22—C21—H21	119.9
C10—C11—C12	107.9 (3)	C21—C22—C17	119.5 (4)
C15—C11—H11	111.8	C21—C22—H22	120.3
C10—C11—H11	111.8	C17—C22—H22	120.3
C12—C11—H11	111.8		
			
O1—S1—C2—C7	−37.9 (4)	C17—S2—C10—C11	19.8 (3)
O2—S1—C2—C7	−167.9 (3)	C9—C10—C11—C15	64.1 (5)
C1—S1—C2—C7	77.0 (3)	S2—C10—C11—C15	−116.0 (4)
O1—S1—C2—C3	145.0 (3)	C9—C10—C11—C12	−47.8 (5)
O2—S1—C2—C3	14.9 (4)	S2—C10—C11—C12	132.1 (3)
C1—S1—C2—C3	−100.1 (3)	C15—C11—C12—C13	−42.9 (4)
C7—C2—C3—C4	0.8 (6)	C10—C11—C12—C13	73.5 (4)
S1—C2—C3—C4	177.9 (3)	C11—C12—C13—C14	44.6 (4)
C2—C3—C4—C5	−1.5 (6)	C11—C12—C13—C16	−68.0 (5)
C2—C3—C4—Cl1	−178.0 (3)	C16—C13—C14—C15	86.1 (5)
C3—C4—C5—C6	0.9 (6)	C12—C13—C14—C15	−30.4 (5)
Cl1—C4—C5—C6	177.3 (3)	C13—C14—C15—C11	2.1 (5)
C3—C4—C5—C8	−174.6 (3)	C10—C11—C15—C14	−86.7 (4)
Cl1—C4—C5—C8	1.8 (5)	C12—C11—C15—C14	28.3 (4)
C4—C5—C6—C7	0.4 (6)	C10—C9—C16—O4	177.3 (4)
C8—C5—C6—C7	176.2 (3)	C8—C9—C16—O4	−1.1 (7)
C5—C6—C7—C2	−1.0 (6)	C10—C9—C16—C13	0.7 (6)
C3—C2—C7—C6	0.5 (6)	C8—C9—C16—C13	−177.6 (4)
S1—C2—C7—C6	−176.6 (3)	C14—C13—C16—O4	106.6 (5)
C4—C5—C8—O3	131.3 (4)	C12—C13—C16—O4	−142.6 (5)
C6—C5—C8—O3	−44.2 (5)	C14—C13—C16—C9	−76.9 (5)
C4—C5—C8—C9	−46.5 (5)	C12—C13—C16—C9	33.9 (6)
C6—C5—C8—C9	138.0 (4)	C10—S2—C17—C18	74.3 (3)
O3—C8—C9—C10	−31.3 (6)	C10—S2—C17—C22	−111.6 (3)
C5—C8—C9—C10	146.4 (4)	C22—C17—C18—C19	0.5 (6)
O3—C8—C9—C16	147.0 (4)	S2—C17—C18—C19	174.5 (3)
C5—C8—C9—C16	−35.3 (5)	C17—C18—C19—C20	−1.0 (6)
C16—C9—C10—C11	5.7 (6)	C18—C19—C20—C21	1.0 (7)
C8—C9—C10—C11	−176.0 (3)	C19—C20—C21—C22	−0.4 (6)
C16—C9—C10—S2	−174.2 (3)	C20—C21—C22—C17	−0.1 (6)
C8—C9—C10—S2	4.1 (5)	C18—C17—C22—C21	0.1 (5)
C17—S2—C10—C9	−160.3 (3)	S2—C17—C22—C21	−174.0 (3)

### Hydrogen-bond geometry (Å, º)

**Table:**

*D*—H···*A*	*D*—H	H···*A*	*D*···*A*	*D*—H···*A*
C1—H1*B*···O4^i^	0.98	2.44	3.322 (4)	150
C1—H1*C*···O4^ii^	0.98	2.52	3.477 (5)	165
C3—H3···O2^iii^	0.95	2.53	3.422 (4)	157

Symmetry codes: (i) *x*, −*y*+1/2, *z*+1/2; (ii) −*x*, *y*−1/2, −*z*+3/2; (iii) −*x*, −*y*+1, −*z*+2.
