# Supplementary figures and images for: Crystal structure of benzobi­cyclon
Source: Acta Crystallogr E Crystallogr Commun. 2015 Dec 9;71(Pt 12):o1035. doi: 10.1107/S2056989015023221 (PMC4719965; doi:10.1107/S2056989015023221)

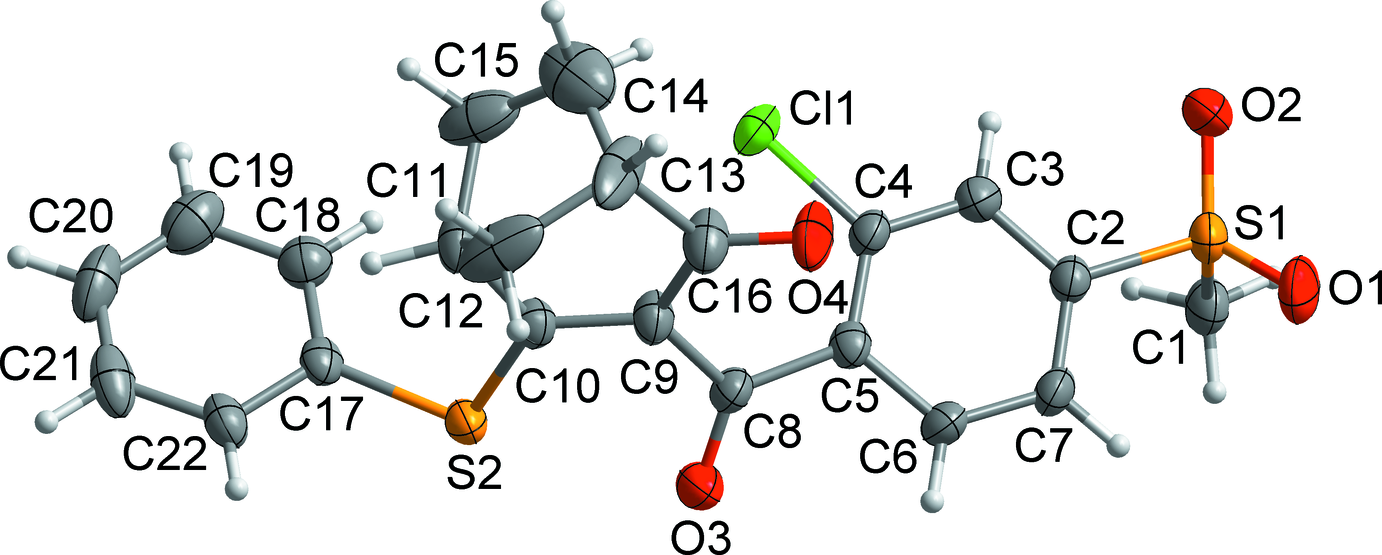

Supplement: Supplementary file 4 [file e-71-o1035-fig1.tif]

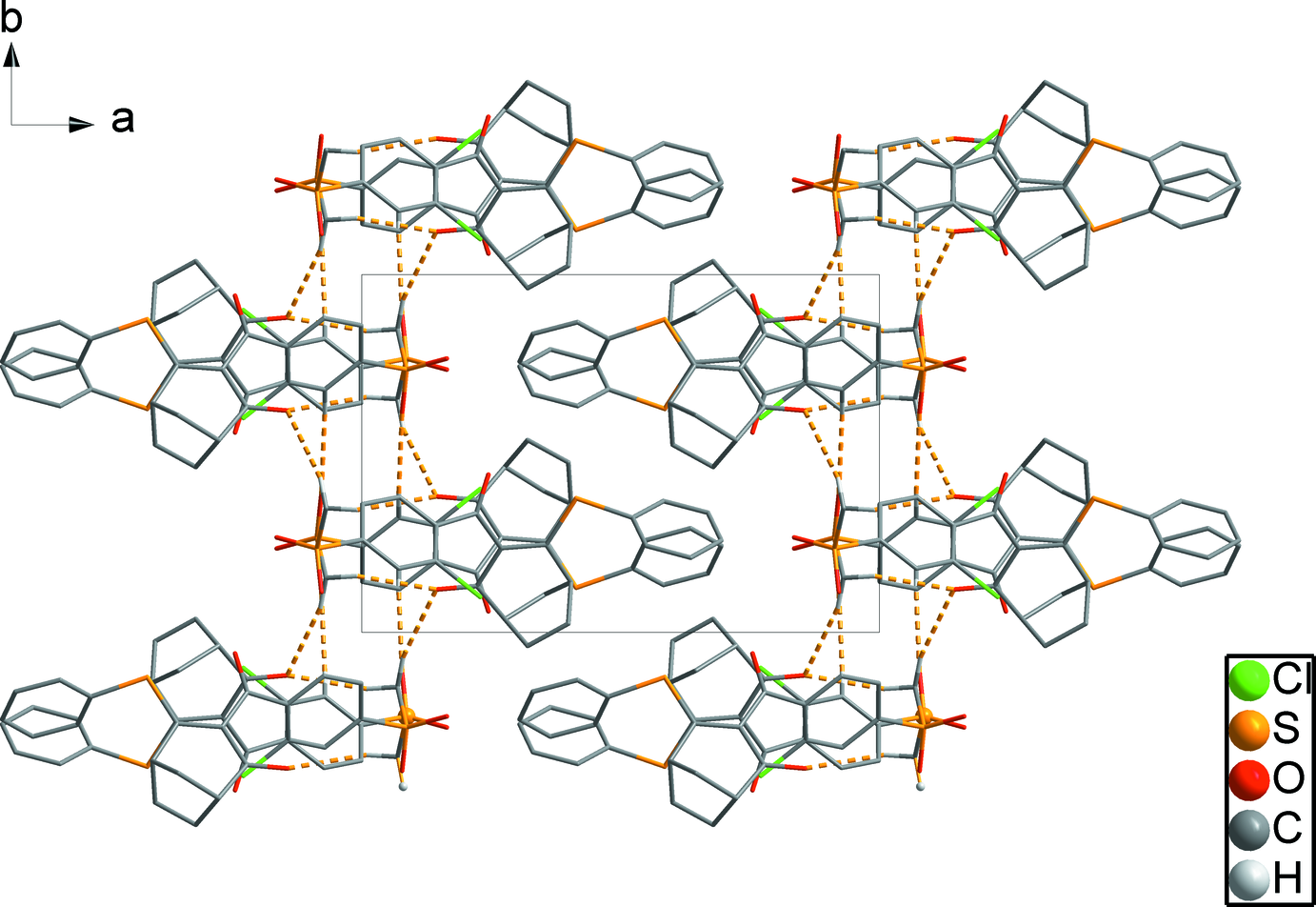

Supplement: Supplementary file 5 [file e-71-o1035-fig2.tif]
